# International Health and the League

**Published:** 1920-11-20

**Authors:** 


### THE PUBLIC WELL-BEING.

### International Health and the League.

### IMPORTANT PRACTICAL PROPOSAL.

Now that the Assembly of the League of Nations is holding its first session at Geneva, it is appropriate that some reference should be made to the work of the *Office International d'Hygiène Publique*, which has within the last few days concluded its autumn session. It is interesting to state, according to definite information which we have received from the Ministry of Health, that the proposal is for the work of the *Office International* to be continued in association with the League of Nations, and that with certain modifications it should form an integral part of the scheme for the International Health Organisation, which is to be submitted by the Council of the League to the Assembly now in session at Geneva.

#### Seamen and Venereal Infection.

The recent conferences, which extended over a period of ten days, were of exceptional interest and importance. Among the several subjects connected with public health that were discussed the question of venereal disease among merchant seamen occupied a prominent place, and considerable time was devoted to a problem that not only concerns the seaman himself, but closely affects the population of maritime and river ports in all countries.

As is now well known, an active campaign has been instituted by the Ministry of Health in this country by the establishment by local authorities of treatment centres for venereal disease in all populous districts, and similar steps have been taken by many health authorities abroad. But the value of such treatment depends, to an extent not generally understood, on its systematic continuance, in some cases for many months; and the merchant seaman, by the conditions of his employment, is frequently prevented from obtaining it. His long absences at sea render him peculiarly liable to contract these diseases on his occasional visits ashore, and to become a means of spreading them at other ports and on his return home.

#### A Regular System.

The *Office International* considered that the matter was of such importance that it was not only advisable, but urgently necessary, that a regular system should be established by international agreement to provide the means by which merchant seamen might obtain continuous treatment for venereal disease at seaports, and it has prepared a draft international agreement on the subject, for submission to the different Governments and to the League of Nations. As the members of the Committee of the *Office International* are experts officially appointed by the Governments of the countries they represent, any recommendations they put forward must of necessity carry great weight.

The proposed international agreement would provide for the establishment of special centres in the chief sea and river ports of all countries at which merchant seamen irrespective of nationality could obtain free and skilled treatment for venereal disease. By means of special confidential forms containing medical notes of diagnosis and treatment to be supplied to the seamen, the doctor at the next port of call would be enabled to continue the treatment with an accurate knowledge of the history of his patient and of the steps that had previously been taken to deal with the case.

The seaman would also be provided, without cost, with such drugs or dressings as may be necessary for him during the voyage from port to port; and, in order to bring the existence of these free-treatment centres to the knowledge of the seamen, and to induce them to take advantage of them, a duty would be laid on ship-masters and port health authorities to inform the crews of all vessels entering a port of the situation of these centres and the hours of consultation.

#### Industrial Tuberculosis.

Many other questions of great social as well as medical importance were discussed at the conferences, including the international aspect of industrial tuberculosis and the hygiene of ships' crews. Considerable time was also devoted to consideration of the revision of the International Sanitary Convention of 1912.

The British delegates put forward a series of important proposals to bring international measures for the prevention of the spread of infectious diseases into accord with recent scientific knowledge and with the new conditions of administration which have arisen since the war.

#### Forty Countries Represented.

The proceedings were of special interest in view of changes that may shortly be brought about in the constitution of the Committee of the *Office International d'Hygiène Publique*. This office was established in 1907 by an international agreement, to which nearly forty different countries are parties, and at the session just concluded thirty States were represented, the British delegates being Dr. G. S. Buchanan (Great Britain), Sir Havelock Charles (India), Dr. A. Granville (Egypt), Dr. Perrin Norris (Australia), Dr. Steegman (Canada), and Dr. P. G. Stock (South Africa).

Very substantial results are to be looked for from the functioning of this widely representative and useful body, in association with the League of Nations.

